# Costing of physical activity programmes in primary prevention: a review of the literature

**DOI:** 10.1186/2191-1991-1-17

**Published:** 2011-10-26

**Authors:** Silke B Wolfenstetter, Christina M Wenig

**Affiliations:** 1 Helmholtz Zentrum München, German Research Center for Environmental Health, Institute of Health Economics and Health Care Management, Ingolstädter Landstraße 1, 85764 Neuherberg, Germany; 2 Ludwig-Maximilians-Universität München, Institute of Health Economics and Health Care Management and Munich Center of Health Sciences, Ludwigstr. 28 RG, 80539 Munich, Germany

**Keywords:** Economics, Costs and Cost Analyses, Motor Activity, Primary prevention, Intervention Studies

## Abstract

This literature review aims to analyse the costing methodology in economic analyses of primary preventive physical activity programmes. It demonstrates the usability of a recently published theoretical framework in practice, and may serve as a guide for future economic evaluation studies and for decision making.

A comprehensive literature search was conducted to identify all relevant studies published before December 2009. All studies were analysed regarding their key economic findings and their costing methodology.

In summary, 18 international economic analyses of primary preventive physical activity programmes were identified. Many of these studies conclude that the investigated intervention provides good value for money compared with alternatives (no intervention, usual care or different programme) or is even cost-saving. Although most studies did provide a description of the cost of the intervention programme, methodological details were often not displayed, and savings resulting from the health effects of the intervention were not always included sufficiently.

This review shows the different costing methodologies used in the current health economic literature and compares them with a theoretical framework. The high variability regarding the costs assessment and the lack of transparency concerning the methods limits the comparability of the results, which points out the need for a handy minimal dataset of cost assessment.

## Introduction

The prevalence of physical inactivity among adults is increasing worldwide. Several diseases such as diabetes mellitus type 2, dyslipoproteinaemia and cardiovascular disease are associated with overweight and physical inactivity [1]; therefore, prevention of physical inactivity is one of the WHO's European regional targets [2]. A positive correlation between physical activity and positive psychological, physiological as well as social effects was found in many reviews and meta-analyses with a focus on secondary prevention. Furthermore, physical activity interventions are shown to be clinically effective [3,4]. Data on the cost-effectiveness of physical exercise intervention programmes is needed to base decisions on possible implementation and transferability on valid information. There are many reviews concerning the cost-effectiveness of secondary prevention programmes that include physical exercise as a treatment option [5,6]. Earlier reviews examined the economic results of preventive physical activity programmes without differentiation of primary and secondary prevention [7-9]. One recent review evaluated the economic evidence and transferability of physical activity interventions in primary prevention. This study concluded that the level of economic evidence as well as the transferability and comparability of cost-effectiveness results are limited because of differences in the methodology used and a lack of transparency [10]. The results of cost-effectiveness studies primarily depend on the cost components included in the calculation. Nevertheless, all of the existing reviews concentrated on the summary of findings and none of the studies analysed the applied costing methodologies in detail.

This present literature review aims to fill this gap by providing an in-depth analyses of the cost assessment of economic analyses of primary preventive physical activity programmes using similar review techniques as in our previous review article [10]. It thereby demonstrates the usability of a theoretical framework which is based on different well established methods and guidelines and specifically adapted for economic evaluations of primary preventive physical activity programmes [11]. Furthermore, the conclusions drawn may serve as a guide for future economic evaluation studies in this field.

## Materials and methods

### Search process

The databases PubMed/Medline were searched for all possible combinations of three groups of terms in order to identify all relevant studies published before December 2009: The first group broadly described different methods of economic evaluation: 'Costs and Cost Analysis' OR 'Economics'. The second group included different terms assigned to physical activity: 'Movement' OR 'Exercise Therapy' OR 'Exercise Test' OR 'Exercise Movement Techniques' OR 'Exercise Tolerance' OR 'Exercise'. The third group contained terms for prevention: 'Prevention and Control' OR 'Primary Prevention' OR 'Health Promotion' OR 'Accident Prevention' OR 'Centres for Disease Control and Prevention (U.S.)'.

Most of the selected MeSH terms are generic terms, each encompassing a set of subordinate search words. Thus, the search for 'cost-benefit analysis', for example, is covered by the search for 'costs and cost analysis' (MeSH). Similarly, 'motor/physical activity' is assigned to the MeSH term 'movement'. Additional searches in the DIMDI, EconLit and Embase databases were carried out analogously. Based on the assessment of the abstracts, a list of relevant papers was derived. Papers were deemed potentially relevant if the outcomes and costs of a primary prevention physical activity programme were evaluated.

### Inclusion and exclusion criteria

Only studies published in peer-reviewed scientific journals in English, Dutch, French and German before December 2009 were considered for this review. This review is limited to trial-based economic analyses of primary research focusing on an adult population. This type of study has a high priority for the German Institute for Quality and Efficiency in Health Care (IQWiG) providing strong and convincing evidence of efficacy [12]. For the purpose of this review, studies based on secondary research, literature-based modelling and literature reviews were excluded, because they are based on cost data from other studies and not on original cost assessment. Reported findings were not included if they were anecdotal and/or not evaluated. The present review is limited to economic analyses reporting the costs or cost-effectiveness of primary prevention programmes based on physical exercise.

### Data extraction and criteria

**Figure 1:**
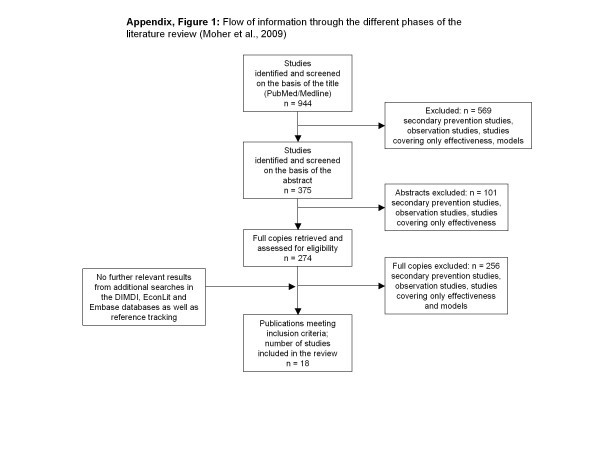
**Flow of information through the different phases of the literature review (Moher et al., 2009)**.

In total, 949 studies resulted from the first search in PubMed, including all studies that were completed before December 2009. Five studies were excluded due to the language limitation. Many of the 944 studies left were secondary prevention studies, observation studies or only covered effectiveness. Others were reviews, focused on children or not peer reviewed, and were thus excluded from further examination. As suggested by the PRISMA-guidelines [13], Figure 1 illustrates the flow of information through the different phases of this literature review. Even though literature search and assessment of the costing methodology followed a systematic approach, this is not a classical systematic review according to PRISMA-guidelines as the focus was rather on highlighting the diversity in cost assessment of existing economic evaluations rather than the assessment of their quality, which has been analysed elsewhere [10]. Eighteen of the finally selected primary research studies described an economic analysis of physical activity programmes for adults. Additional searches in the DIMDI, EconLit and Embase databases showed no further relevant results. Data extraction regarding cost assessment methodology follows a previously published theoretical framework for economic evaluation of physical activity programmes. Data extraction was undertaken and checked by two researchers individually reaching agreement after discussion in all 18 studies.

### Study characteristics and key economic findings

All the 18 studies included were briefly described regarding important characteristics, including 'type of physical exercise intervention, comparator, length of intervention, data collection, study population, country, setting, year(s) of the study, study design, type of economic evaluation'and key economic findings. In order to facilitate comparisons across studies, costs were converted to Euros using purchasing power parities (PPP) [14] if available. These results were inflated to 2008 prices using general price indices (GDP) [15]. In case the information on the base year for prices was missing, the year of the intervention was assumed instead, if indicated.

### Cost assessment

The cost assessment of this review refers to a conceptual framework developed by Wolfenstetter [11] which is based on different well established methodological guidelines and specifically adapted for economic evaluations of primary preventive physical activity programmes. According to this framework, the cost dimension include programme development costs and programme implementation costs (consisting of recruitment costs, programme costs and time costs of participants), and cost savings due to health effects of the intervention. These cost savings consist of direct medical costs, direct non-medical costs and indirect costs.

Programme development costs include costs for initiating and developing a physical activity programme. The importance of this cost category greatly depends on the aims of the decision-maker, for example whether the whole programme had to be adapted to a different target group and/or setting.

The second cost category comprises the programme implementation costs, which include personnel and non-personnel costs resulting from the intervention programme and the recruitment of participants as well as participant time costs. Recruitment costs contain costs that are linked to the recruitment of participants, for example marketing and advertising activities. These activities are considered in the health economic evaluation in terms of, for example, personnel time costs, costs for posters, flyers or a pilot workout. Most studies are economic evaluation of trials. However, the recruitment costs included should mimic the costs of recruiting people for the programme in a real world setting as far as possible.

The programme costs are costs directly associated with the consumption of resources necessary for carrying out the programme and include, for example, personnel expenditures for instructors and trainers, non-personnel costs, like for sports equipment or costs for the gym. The programme related time costs of participants should be analysed and valued according to the principle of opportunity cost. Valuation should depend on whether the time for physical exercise replaces leisure time or labour time. Similar to productivity losses due to illness, lost labour time due to participation in prevention programmes could be valued using the human capital or friction cost approach. Yet, research protocol driven participant time costs should not be included because time spent in a research study will differ from time spend for participation in a real community physical activity programme.

The incremental cost-effectiveness ratio is calculated based on the resulting net costs and the health effect of the programme.

The cost savings are composed of direct medical costs, direct non-medical costs and indirect costs depending on the chosen perspective. Although a societal perspective requires the inclusion of all direct and indirect costs, the company perspective might focus on indirect costs resulting from sick leave, and the healthcare payer perspective on the cost components they have to reimburse, primarily direct medical costs. Direct medical costs are costs associated directly with the utilisation of healthcare services, for example physician contacts, medication, hospitalisation, rehabilitation, remedies, aids and also over-the-counter medication. They can also include patients' out-of-pocket expenses. The level of aggregation of the costs also depends on the availability of data on costs. Direct non-medical costs include costs such as expenditures for additional health programmes, costs of transportation or information costs.

Indirect costs comprise costs of illness-related absenteeism from paid work (short- and long-term absence from work) as well as from unpaid work (e.g. housework), and costs of productivity loss or gain due to morbidity or premature mortality. Indirect costs will only be included if a societal or company perspective is chosen.

Health effects of health promotion programmes and a corresponding cost reduction could occur with a long time delay. Most individuals appear to have a positive rate of time preference, i.e. a preference to enjoy benefits today more than in the future and, conversely, favour paying costs in the future rather than today. Thus, Smith and Gravelle recommended the need for discounting if the evaluation takes more than 18 months [16]. The practice of the chosen discount rate depends on country-specific recommendations [12,17,18].

A high level of detail in reporting of resource use has to be aimed for as well as exact description of the valuation methods.

This article presents an overview of the different cost categories that were assessed in the 18 reviewed studies. Additionally, important methodological issues such as price year and valuation method, presentation of physical units, perspective, discount rate and the existence of a sensitivity analysis are presented.

## Results

### Study characteristics and key findings

Altogether, 18 economic analyses of physical activity programmes in primary prevention from seven different countries (Taiwan, UK, New Zealand, Netherlands, Canada, USA and Australia) were identified. All were published in English between 1982 and 2008. Table 1 summarises the study characteristics and Table 2 offers an overview on the key economic findings.

**Table 1:** Study characteristics

Type of econ. analysis	Author (year of publication)	Type of physical exercise intervention	Length of intervention (data collection)	Comparator	Health Outcome	Number of participants (sex), age (years)	Country, setting, study design
CUA	Chen et al. (2008) [34]	Walking	12 weeks (baseline-12 weeks)	no intervention	QALY	98 (m/f), > 65	Taiwan, community, RCT
	
	Munro et al. (2004) [23]	Free exercise classes by qualified exercise leader	2 years (baseline-1 year - 2 years)	usual care	Mortality, health status, QALY	6,420 (m/f), > 65	UK, community, Cluster RCT

CEA	Elley et al. (2004) [20]	Green prescription: verbal and written exercise advice by GP and telephone exercise specialist	1 year (baseline-1 year)	usual care	Total energy expended (change in PA), QALY	878 (m/f), 40-79	New Zealand, GPP, Cluster RCT
	
	Stevens et al. (1998) [22]	Individual PA by exercise development officer	10 weeks (baseline-10 weeks- 8 months)	EI vs MI	PA, number of sedentary people	714 (m/f), 45-74	UK, GPP, RCT
	
	Robertson et al. (2001a) [27]	Individually home-based PA by district nurse	1 year (baseline-1 year)	usual care	Falls and injuries	240 (m/f), ≥ 75	New Zealand, GPP, RCT
	
	Robertson et al. (2001b) [26]	Individually home-based PA by general practice nurse	1 year (baseline-1 year)	usual care	Falls and injuries	450 (m/f), ≥ 80	New Zealand, GPP, CT
	
	Robertson et al. (2001c) [25]	Individually home-based PA by physiotherapist	2 years (baseline-2 years)	usual care	Falls and injuries	233 (f), ≥ 80	New Zealand, GPP/home, RCT
	
	Proper et al. (2004) [21]	Worksite PA counselling	9 months (baseline-9 months)	EI vs MI	Sick leave, PA, cardiovascular fitness	299 (m/f), 44	Netherlands, municipal services, RCT
	
	Shephard (1992) [35]	Employee fitness programme	12 years (6 months- 18 months- 7 years-10 years-12 years)	no intervention	PA, absenteeism; corporate commitment	534 (m/f), age n.s.	Canada, company, CT
	
	Sevick et al. (2000) [36]	Structured exercise intervention and supervised behavioural skills training	2 years (baseline-6 months-2 years)	no intervention	Energy expenditure (kcal/gk/day), peak flow (VO2 in ml/kg/min); PA; heart rate, blood pressure, weight	235(m/f), 35-60	USA, company, RCT
	
	Finkelstein et al. (2002) [24]	WISEWOMAN project: screening and counselling	1 year (baseline-1 year)	MI vs EI	Risk of CHD, LYG	1586 (f), 40-64	USA, community/healthcare sites, RCT
	
	Dzator et al. (2004) [19]	Self-directed intervention of PA and nutrition delivered by mail (low level) or by mail and group sessions (high level)	16 weeks (baseline-16 weeks-1 year)	no intervention	BMI, Total/HDL cholesterol, blood pressure, PA (W/kg), nutrition fat intake	137 (m/f) couples, all ages	Australia, home, RCT
	
	The Writing Group (2001) [43]	PA counselling with current recommended care	2 years (baseline- 6 months- 1 year -18 months - 2 years)	usual care	Cardio-respiratory fitness, self-reported PA	874 (m/f), 35-75	USA, GPP, RCT

other Econ. Analysis	Ackermann et al. (2003) [33]	Group-based exercise community programme	20.7 months (baseline-20,7 months)	no intervention	Endurance, strength, balance, flexibility	4,456 (m/f) ≥ 65	USA, community, Retro MCT
	
	Ackermann et al. (2008) [28]	Group-based PA programme	2 years (baseline-1 year-2 years)	no intervention	Comorbidity (RxRisk-score, lipo-protein, cholesterol, triglycerides, haemoglobin, DM, CAD, arthritis)	1,188 (m/f), ≥ 65	USA, community, Retro MCT
	
	Baun et al. (1986) [32]	Unsupervised and supervised health and fitness activities	1 year (baseline-1 year)	no intervention	Absenteeism rates	517 (m/f), ≥ 55	USA, company, RCT
	
	Shephard (1982) [31]	Employee fitness and lifestyle programme	9 months (baseline-1 year-2 years)	no intervention	-	534 (m/f), 21- < 90	Canada, company, CT
	
	Shephard et al. (1983) [30]	Employee fitness programme	9 months (baseline-9 months)	no intervention	Fitness, HHA- score	326 (m/f), 30.5-37.9 (mean)	Canada, company, RCT

Abbreviations: CAD: cardiocascular disease; CEA: cost-effectiveness analysis; CT: controlled trial; CUA: cost-utility analysis; DM: Diabetes Mellitus; Econ.: economic; EI: enhanced intervention; f: female; GP: general practitioner; GPP: general practitioner practices; m: male; MCT: matched controlled trial; MI: minimum intervention; n.s.: not stated; PA: physical activity; RCT: randomised controlled trial; Retro: retrospective; UK: United Kingdom; USA: United States of America.

**Table 2:** Key economic findings

Type of econ. analysis	Author (year of publication)	Key economic findings (costs as reported in studies)	Reported costs (or costs per effectiveness-outcome) converted to 2008 EUROS
CUA	Chen et al. (2008) [34]	ICER: USD15,103/QALY gained	[No year of intervention]
	
	Munro et al. (2004) [23]	(1) ICER: EUR17,172/QALY gained; (2) CE: EUR4,739-EUR32,533/QALY	(1) EUR18,364 (2) EUR5,068-EUR34,791

CEA	Elley et al. (2004) [20]	(1) Monthly CER: NZD11/kcal/kg/day; (2) ICER: NZD1,756 converted sedentary adult to an active state in 12 months	(1) EUR 8 (2) EUR1,268
	
	Stevens et al. (1998) [22]	(1) GBP623/one sedentary person doing more PA; (2) GBP2,498/moving someone who is active but below min. level	[No year of intervention]
	
	Robertson et al. (2001a) [27]	(1) ICER: NZD1,803/fall prevented; (2) NZD7,471/injurious fall prevented (cost saving for people older than 80 years)	(1) EUR1,423 (2) EUR5,898
	
	Robertson et al. (2001b) [26]	(1) ICER: NZD1,519/fall prevented; (2) NZD3,404/injurious fall prevented (exercise programme only more cost-effective for those over 80 years)	(1) EUR1,202 (2) EUR2,694
	
	Robertson et al. (2001c) [25]	(1) ICER: NZD314/fall prevented (1 year); NZD265/fall prevented (2 years); (2) NZD457/injurious fall prevented (1 year); NZD426/injurious fall prevented (2 years)	(1) EUR261; EUR220 (2) EUR379; EUR353
	
	Proper et al. (2004) [21]	CER without (with) imputation of effect data: (1) EUR5 (EUR3)/extra energy expenditure (kcal/day); (2) EUR235 (EUR46)/beat per minute decrease in submaximal heart rate; (3) total net costs (9 months): EUR305; (4) benefits from sick leave reduction (1 year later): EUR635	(1) EUR6 (EUR3) (2) EUR267 (EUR52) (3) EUR346 (4) EUR721; [Apy 2000]
	
	Shephard (1992) [35]	(1) Programme benefits/worker/year (participation rate of 20%): CAD679; (2) ROI: CAD7; (3) long-term cost-benefit: CAD5 to 1	(1) EUR757 (2) EUR8 (3) EUR5 to 1
	
	Sevick et al. (2000) [36]	(1) Lifestyle intervention (24 months): USD20/additional kcal/kg/day per month (2) Structured intervention (24 months): USD71/additional kcal/kg/day per month (different outcomes)	(1) EUR23 (2) EUR81; [Apy 1998]
	
	Finkelstein et al. (2002) [24]	(1) IC of EI per person: USD191; (2) ICER: USD637/1% point additional decrease in 10-year probability of CHD for EI compared with MI; (3) nearly USD5,000/LYG (n.sig.)	(1) EUR226 (2) EUR753 (3) EUR5,911; [Apy 1996]
	
	Dzator et al. (2004) [19]	1-year follow-up: Average incremental costs/unit change in outcome variables: (1) high intervention: AUD460; (2) low intervention: AUD459; (3) control: AUD462 (different outcomes)	[No year of intervention]
	
	The Writing Group (2001) [43]	(1) For 2 years: IC/participant of assistance intervention: USD500; (2) IC of counselling intervention/participant: USD1,100	(1) EUR591 (2) EUR1,300; [Apy 1996]

other Econ. Analysis	Ackermann et al. (2003) [33]	(1) Increase in annual healthcare costs: USD642 (IG) and USD1,175 (CG); (2) Savings in annual healthcare costs: USD533	(1) EUR735 and EUR1,345 (2) EUR610; [Apy 1998]
	
	Ackermann et al. (2008) [28]	Adjusted total healthcare costs (after 2 years): USD1,186 lower	EUR1,115
	
	Baun et al. (1986) [32]	(1) Healthcare costs: USD553 (participants) and USD1,146 (controls); (2) Healthcare savings: USD593	(1) EUR921and EUR1,908 (2) EUR987
	
	Shephard (1982) [31]	Savings per employee/year: CAD84.50 (ICER n.s.)	-
	
	Shephard et al. (1983) [30]	Decrease in body fat related to increased hospital utilisation and medical care costs in men and women (no $ values reported) (different outcomes)	[no $ values reported]

Abbreviations: apy: assumed price year; CAD: Canadian dollars; CE: cost-effectiveness; CEA: cost-effectiveness analysis; CER: cost-effectiveness ratio; CHD: cardiovascular heart disease; CG: control group; CUA: cost-utility analysis; Econ.: economic; EI: enhanced intervention; EUR: Euro; GBP: Great Britain Pound; ICER: incremental cost-effectiveness ratio; IG: intervention group; kcal: kilocalorie; LYG: life years gained; MI: minimum intervention; min: minimum; n.s.: not stated; NZD: New Zealand Dollars; PA: physical activity; QALY: quality-adjusted life year; ROI: return on investment; kg: kilogram; USD: US dollars.

There was a great variation in the type (e.g., supervised and unsupervised physical activity) and length (10 weeks-12 years) of physical exercise programme as well as the adult study populations (e.g., all ages or 80 years and older) in the reviewed interventions. The outcomes varied from specific measures, for example activity change or health events (falls), to generic measures, such as quality-adjusted life-years (QALYs) or disability-adjusted life-years (DALYs). Moreover, the authors of the analysed studies considered different types of economic analyses. Owing to different outcome parameters, the comparison of the results between studies is not possible in all cases. To facilitate comparison of the study results Tables 1 and 2 are organised first according to the type of economic evaluation and second according to the central outcomes.

### Cost assessment

Programme development costs have only been itemised in two of the 18 studies [19,20] and mentioned in one [21]. Recruitment costs were explicitly assessed and disclosed in three studies in terms of, for example, invitations, reminders and marketing (TV/newspaper) [22-24]. Robertson et al. included recruitment costs in total programme costs [25-27] and one further study only mentioned these costs [21]. Programme costs were explicitly disclosed in all but six studies [28-33]. The contents of the programme costs vary considerably, primarily depending on the accuracy of the reporting and the type of programme.

Chen et al. included lost income for the participant and his/her companion due to the intervention [34]. Two studies valued these costs as zero [26,27]. As most studies did not include this component, they apparently assumed exercise to be part of leisure time.

Direct medical costs were included in nine studies [20,25-28,30-33] predominantly appropriate to their chosen perspective if stated. Direct non-medical costs were only collected by one study in terms of costs of additional exercise [20]. Five studies assessed sick leave days or hours [20,21,23,32,35], but only two cost studies calculated indirect costs appropriate to their chosen perspective, the societal or company perspective [20,21]. Robertson and colleagues have chosen a societal perspective and did not include direct non-medical costs as well as indirect costs in their calculation, as all their participants were older than 75 years [25-27]. The contents of health savings vary greatly among the reviewed studies, primarily depending on the perspective, but also on the availability of data, the study population and the accuracy of the reporting.

### Important methodological aspects

Three of the reviewed studies discounted future costs with a 5% rate according to their time of intervention including the follow-up period [19,20,36]. Six studies evaluated a physical activity programme over a period of 18 months and discounted neither costs nor effects [23,25,28,29,33,35].

A separate and transparent presentation of how the quantities of resource use were determined was found in more than half the reviewed studies, which improves the traceability of the cost assessment. In many studies, the physical units consumed are monetised with market prices reflecting opportunity costs, and personnel time was valued by average wage rates as recommended [37,38]. Other studies refer to financial records and statistics, for example from insurances or from hospitals for cost estimation [25,30-32]. One study did not reveal the methodology of valuation at all [29]. All costs were declared in their own country's currency. Eight studies did not explicitly state the price year of adjustment [19,21,22,24,29,33,34,36], which impedes the transferability of the results. Sensitivity analysis can be used to examine the uncertainty related to key assumptions in the calculation of costs, for example in calculating different rents for gyms or tariffs for physical exercise trainers [39]. Seven studies did not conduct sensitivity analyses for the costs or the effects of the intervention under review [29-35]. The assessments of all cost categories and methodological aspects are summarised in Table 3.

**Table 3:** Costing in economic analyses of physical activity programmes in primary prevention

Type of econ. Ana-lysis	Author (year)	PDC components	Programme implementation cost components			Savings due to health effect (cost components)			Methods				
			
			Recruit-ment	Programme	Participant time	Direct medical	Direct non-medical	Indirect	Pers-pective	Phys.units	d (%)	SA	Price year/valuation of cost components
CUA	Chen et al. (2008) [34]	-	-	personnel, paper, machine maintenance, transport, extra equipment, babysitter	lost income	- (hospital, outpatient and emergency visits)^a^	-	-	n.s.	+/-	-	-	n.s./personnel: salary
	
	Munro et al. (2004) [23]	-	reminders, invitation, leaflet	admin., rent (office, halls), travel, personnel, consumables	-	- (hospital, outpatient, emergency, GP)^a^	-	- (morbidity, mortality)^a^	hcp	+	-	+	2003/04/actual prices paid

CEA	Elley et al. (2004) [20]	set-up and coordinating	-	coordinating, sports foundation support, staff training, personnel, admin., rent, printing, postage	-	health funder/patient costs: accident-related referrals, GP visits, hospitalisation	costs for add. exercise	sick leave	soc/hcp/pat	+	5 c	+	2001/personnel, overhead, productivity loss: average wages, GP: average consultation charges; therapists: average patient surcharge; hospital costs: local district health board
	
	Stevens et al. (1998) [22]	-	postage, stationery, admin.	postage, stationery, personnel incl. institution cost, equipment	-	-	-	-	n.s.	-	-	+	n.s./personnel: wage costs plus institution costs
	
	Robertson et al. (2001a) [27]	-	incl. in PC	overhead, personnel, materials, travel, accommodation, postage, pager, admin., equipment, exercise instructor excl.	zero (leisure time)	hospital (emergency room, theatre, ward, physician, radiology, laboratory, blood services, pharmacy products, social workers, physiotherapy, occupational therapy) incl. overhead costs	-	- retired	soc	+	-	+	1998/opportunity costs/overhead cost as 21.9% of observed resource use; physician: average time cost, PIC: hospital and trial records, 1/2 recruitment cost because of control group
	
	Robertson et al. (2001b) [26]	-	incl. in PC	overhead, personnel, materials, travel, accommodation, postage, pager, admin., equipment, exercise instructor excl.	zero (leisure time)	hospital (emergency, theatre, ward, physician, radiology, laboratory, blood services, pharmaceuticals, social workers, physiotherapy, occupational therapy) incl. overhead	-	- retired	soc	+	-	+	1998/opportunity costs/overhead cost as 21.9% of observed resource use; physician: average time cost, PIC: hospital and trial records, 1/2 recruitment cost because of control group
	
	Robertson et al. (2001c) [25]	-	incl. in PC	overhead, personnel, materials, equipment	-	hospital (inpatient, outpatient, emergency, overhead), home care, GP, medical specialist, dentist, out-of-pocket expenses	-	- retired	soc	+	-	+	1995/fall-associated hospital costs: hospital financial records; physicians: Statistics NZ; out-of-pocket expenses: patient report
	
	Proper et al. (2004) [21]	m	m	information session, physician/counsellor consultation, written information, fitness/health test, personnel	-	-	-	sick leave	comp	-	-	+	n.c.s./fair market value; mean salary costs of civil servants
	
	Shephard (1992) [35]	-	-	gym, equipment, rent, operating/maintenance cost (consulting, personnel, interior construction), contribution/membership fees	-	(hospital and medical claims)^b^	-	(absenteeism, productivity)^b^	comp	-	-	-	1990/opportunity costs
	
	Sevick et al. (2000) [36]	-	-	personnel, computerised tracking system, materials, printing, postage, facilities, health club memberships	-	-	-	-	hcp, cp	-	5 c	+	n.s./actual price; personnel: hourly wage rate incl. fringe rate
	
	Finkelstein et al. (2002) [24]	-	newspaper, TV	personnel, equipment and supplies, admin.	-	-	-	-	n.s.	+/-	3 e	+	n.c.s./fair market values; services below market value: rates for similar services
	
	Dzator et al. (2004) [19]	PD, review, improvement	-	personnel, printing, postage, equipment, information package, consumables, rent	-	-	-	-	n.s.	+	5 c	+	n.s./equipment annuitised; resource use: corresponding unit cost; time of staff: wage rate
	
	The Writing Group (2001) [43]	-	-	- (individual visits by GP, telephone calls, classes, newsletters)^b^	-	-	-	-	n.s.	+/-	-	-	n.s./valuation n.s.

Other Econ. Ana-lysis	Ackermann et al. (2003) [33]	-	-	- (admin., charge per enrolee per visit)^a^	-	hospital, primary care/preventive services (staff, pharmacy, laboratory, radiology, inpatient, community services, overhead)	-	-	n.s.	+	-	-	n.s./units of service weighted by (technical) relative value units, College of Anatomical Pathology units, visits length
	
	Ackermann et al. (2008) [28]	-	-	- (charge per enrolee per visit)^a^	-	hospital, primary care, speciality care, (staff, nursing, pharmacy, laboratory, radiology, hospital inpatient, community services, overhead)	-	-	n.s.	+	-	+	2005/units of service weighted by (technical) relative value units, College of Anatomical Pathology units, visits length
	
	Baun et al. (1986) [32]	-	-	- (fitness centre equipment)^a^	-	inpatient and outpatient costs	-	- (absenteeism)^a^	hcp	+	-	-	1983/claims records from insurance
	
	Shephard (1982) [31]	-	-	-	-	hospital, medical claims (electrocardiography, orthopaedic, obstetric/gynaecology services, other)	-	-	n.s.	+	-	-	1977/78/Ontario Health Insurance Plan records
	
	Shephard et al. (1983) [30]	-	-	-	-	hospital, total medical claims, other	-	-	n.s.	+/-	-	-	1977/78/Ontario Health Insurance Plan records

^a ^Utilisation components were mentioned but not monetised or included in the cost calculation.

^b ^Unclear which of the given utilisation components are included in the total costs.

Abbreviations: add.: additional; admin.: administration; CEA: cost-effectiveness analysis; c: costs; comp: company; cp: clinician perspective; CUA: cost-utility analysis; d: discounting; e: effects; Econ.: economic; excl.: exclusive; GP: general practitioner; hcp: healthcare payer; incl.: inclusive; m: mentioned but not explained in detail; n.c.s.: not clearly stated; n.s.: not stated; NZ: New Zealand; pat: patient; PC: programme costs; PDC: programme development costs; phys: physical; SA: sensitivity analysis; soc: societal.

The problems of comparing economic evaluations of primary prevention programmes mainly refer to the intervention and its context specific aims as well as the purpose of the decision-maker and his/her options. The decision-maker determines the perspective, which has to be chosen carefully and stated explicitly, as it defines the cost categories that have to be included in the cost analysis. The patient perspective reduces the relevant costs to out-of-pocket expenses and lost time in both programme costs (e.g. programme fees, lost leisure time) and savings (e.g. out-of-pocket expenses for pharmaceuticals, indirect costs regarding unpaid work). Only Elley et al. considered the patient perspective next to the healthcare payer and societal perspective in their calculations [20]. The indirect costs due to absenteeism are the main savings resulting from health effects from a company perspective, which was chosen in two analyses [21,35]. Both studies included programme costs and examined the costs of sick leave. The healthcare payer perspective was solely chosen by three studies, which would require the inclusion of programme implementation costs as well as direct (non)-medical costs that have to be reimbursed by health insurance. Baun et al. only regarded the direct medical costs compared with no intervention. Sevick et al. only considered programme costs and did not include direct medical costs even though they took a healthcare payer and provider perspective [32,36]. Munro et al. include both categories in their calculation [23]. The most recommended societal perspective requires a comprehensive assessment of programme implementation costs and all categories of savings due to health effects. Only four of the reviewed studies chose the societal perspective [20,25-27]. Thus, they include healthcare savings as well as detailed programme implementation components. Nine studies did not clearly state their chosen perspective and only included parts of the cost components. Even if most studies did provide at least a rough description of included cost components, the level of detail differed substantially, for example equipment or administration and what it included. Table 4 presents an overview of recommendations for the minimal basic datasets depending on the chosen perspective. The single cost items refer to the detailed description in the 'materials and methods' section and in Wolfenstetter 2011 [11].

**Table 4:** Minimal basic datasets depending on the chosen perspective

Cost components		Perspective			
		
		Societal	**Healthcare payer**^**1**^	Company	Patient
Programme development (if programme has to be adapted to setting and population)		+	+	+	
Programme implementation cost components	Recruitment	+	+	+	
	
	Programme	+	+	+	
	
	Participant time	+	+	+	+

Savings due to health effect	Direct medical	+	+^**2**^		+^**3**^
	
	Direct non-medical	+	+^**2**^		+^**3**^
	
	Indirect	+		+	

^1^Depending on the country-specific healthcare system and reimbursement policies of the insurance.

^2^Out-of-pocket expenses excluded.

^3^Only out-of-pocket expenses.

## Discussion and conclusion

In sum, 18 international economic analyses of primary preventive physical activity programmes were identified and analysed regarding their key economic findings and their costing methodology. Most of the reviewed studies deduce that the investigated intervention is good value for money compared with alternatives or even cost saving. However, these results are difficult to compare, mainly because of methodological differences, for example the type of economic evaluation, regarded outcomes, included cost components (depending on the chosen perspective) or the valuation of utilisation.

As the inclusion of cost variables such as for gym hire, equipment and the salaries of site health personnel are not standardised, decision-makers confronted with the question of whether or not to transfer and implement the programme need to be fully informed about the cost items included in the total programme costs. For the economic evaluation of physical activity programmes not only components of the programme costs, but also potential savings due to health effects (i.e. direct and indirect costs) should be included in the costs calculation. For the assessment of all cost components, it is also important that the utilisation in physical units as well as the methodology of valuation are described in detail. Even if most studies did provide a detailed description of the costs of the intervention programme in their country currency, data on the underlying quantities of resources used, discounting/inflation methods and the price year were often not displayed, thus making comparability difficult. Sensitivity analyses should be calculated to clarify uncertainty related to key assumptions. However, the main areas of uncertainty were often not considered in the studies, or sensitivity analyses were of low quality, for example insufficient explanation was given for the range of parameters chosen for the sensitivity analysis.

Costs and cost savings also depend on the time horizon of the evaluation. In the case of physical activity programmes, savings of health service resources emerge as a consequence of reductions in inactivity-related diseases and mortality, leading to a lower utilisation of healthcare services and lower productivity losses [40]. Furthermore costs and cost savings can differ between age-groups and gender, for example costs of productivity losses. This should be considered depending on the chosen study population.

Of course, medical outcome parameters are often more important than the costs of a programme. However, in times of budget restraints, costs gain more and more importance.

The results of cost-effectiveness studies primarily depend on the cost components included and the comparability of the results is difficult if cost assessment differs substantially. Although an earlier review evaluated the economic evidence and transferability of physical activity interventions in primary prevention [10], to our knowledge, none of the already existing reviews in this area analysed the applied costing methodologies in detail. This present literature review aims to fill this gap by providing an in-depth analysis of the cost assessment of economic analyses of primary preventive physical activity programmes as well as a minimal dataset for cost assessment depending on the chosen perspective as a practical guide for the economic evaluation of physical activity programmes. Further methodological problems and more detailed recommendations for the economic evaluations of primary preventive physical activity programmes are comprehensively explained and discussed elsewhere [7,10,41].

The main limitations of this review are that the search was limited to those publications referenced in the given databases; only English, German, French and Dutch papers were considered. However, this excluded only three Japanese and two Spanish studies listed in PubMed. The selection and analysis of the studies was conducted by only two researchers reaching concordance after discussion in all cases but still leaving room for a possible bias. The costs of the studies were adjusted to Euros (2008), if possible, to show better comparability between the study results. However, the explanatory power is limited because country-specific healthcare systems, their prices and charges, etc. were neglected in this calculation.

This review targets clinicians, behavioural scientists, researchers working in the field of public health and decision-makers. It may, to some degree, demonstrate the difficulties of economic evaluation in the area of primary prevention. It aims to provide useful information for researchers, asking which perspective has to be chosen and which cost components have to be assessed for the evaluation to provide an optimal database for decision-making.

There is a gap between theoretical guidelines for cost assessment and their application in practice. One reason is that the chosen costing methods are often greatly dependent on the available data. This review shows that there is little standardisation of what constitute costs in such interventions and their evaluations. The comparability of the cost-effectiveness results of physical activity programmes is problematic on different levels: first, the examined programmes vary considerably in their aims and characteristics suited to their specific context and study population. Second, the methodologies used are often not revealed transparently and, third, if comprehensibly described, the methods and accuracy of reporting differ substantially. In order to generalise the results to other settings, regions or countries, a country-specific adaptation is necessary to account for, for example, different inactivity prevalence, healthcare system characteristics and absolute and relative prices. Recommendations for transferability of study results are given by Welte et al. [42].

In general, the high variability of the costing methods between the studies limits comparability and generalisability. However, the need to identify cost-effective or cost-saving prevention programmes and to transfer study results from one region or country to another is growing. To improve the standardisation and comparability of economic evaluations among different physical activity programmes and among countries, high methodological quality and explicit reporting of a minimal dataset are important, which is a big challenge for health economists.

## Conflict of interests

The authors declare that they have no competing interests.

## Authors' contributions

The databases PubMed/Medline were searched by SBW for all possible combinations of three groups of terms in order to identify all relevant studies published before December 2009. Data extraction and assessment were undertaken and checked by SBW and CMW. SBW and CMW analysed the data and interpreted the results. Both authors drafted the manuscript, read and approved the final version of the manuscript.

## References

[B1] Konig D Bonner G Berg A [The role of adiposity and inactivity in primary prevention of cardiovascular disease] Herz 2007 32 553 559 10.1007/s00059-007-3019-7 17972028

[B2] Physical activity World Health Organization (WHO): Global Strategy on Diet, Physical Activity and Health. http://www.who.int/dietphysicalactivity/en/

[B3] Karmisholt K Gyntelberg F Gotzche PC Physical activity for primary prevention of disease. Systematic reviews of randomised clinical trials Dan Med Bull 2005 52 86 89 16009052

[B4] Gardner MM Robertson MC Campbell AJ Exercise in preventing falls and fall related injuries in older people: a review of randomised controlled trials Br J Sports Med 2000 34 7 17 10.1136/bjsm.34.1.7 10690444 PMC1724164

[B5] Avenell A Broom J Brown TJ Poobalan A Aucott L Stearns SC Smith WC Jung RT Campbell MK Grant AM Systematic review of the long-term effects and economic consequences of treatments for obesity and implications for health improvement Health Technol Assess 2004 8 iii iv 1-182 15147610 10.3310/hta8210

[B6] Kouris-Blazos A Wahlqvist ML Health economics of weight management: evidence and cost Asia Pac J Clin Nutr 2007 16 Suppl 1 329 338 17392129

[B7] Hagberg LA Lindholm L Is promotion of physical activity a wise use of societal resources? Issues of cost-effectiveness and equity in health Scand J Med Sci Sports 2005 15 304 312 10.1111/j.1600-0838.2004.00415.x 16181254

[B8] NICE Rapid review of the economic evidence of physical interventions National Institute for Health an Clinical Excellence (NICE): Rapid review of the economic evidence of physical interventions http://http:/ /www.nice.org.uk/nicemedia/pdf/Physical_Activity_Economic_Review_A pril2006.pdf

[B9] Shepard RJ Current Perspectives on the Economics of Fitness and Sport with Particular Reference to Worksite Programmes Sports Med 1989 7 286 309 10.2165/00007256-198907050-00002 2500691

[B10] Wolfenstetter SB Wenig CM Economic Evaluation and Transferability of Physical Activity Programmes in Primary Prevention: A Systematic Review Int J Env Res Public Health 2010 7 1622 1648 10.3390/ijerph7041622 20617050 PMC2872359

[B11] Wolfenstetter S Conceptual framework for standard economic evaluation of physical activity programmes in primary prevention Prevention Science 2011 Online First 19 Juli 2011 10.1007/s11121-011-0235-4 21773728

[B12] Institute for Quality and Efficiency in Health Care (Institut für Qualität und Wirtschaftlichkeit im Gesundheitswesen (IQWiG)) Allgemeine Methoden, Entwurf für Version 3.0 vom 15.11.2007 [in German] Book Allgemeine Methoden, Entwurf für Version 3.0 vom 15.11.2007 [in German] 2007 City: Institut für Qualität und Wirtschaftlichkeit im Gesundheitswesen (IQWiG) (Editor ed.^eds.)

[B13] Moher D Liberati A Tetzlaff J Altman DG Preferred reporting items for systematic reviews and meta-analyses: the PRISMA statement J Clin Epidemiol 2009 62 1006 1012 10.1016/j.jclinepi.2009.06.005 19631508

[B14] Purchasing Power Parities (PPP) Organisation for Economic Co-operation and Development (OECD): Purchasing Power Parities (PPP). http://www.oecd.org/department/0,3355,en_2649_34357_1_1_1_1_1,00.html

[B15] Consumer Prize Indices Organisation for Economic Co-operation and Development (OECD): Consumer Prize Indices. http://stats.oecd.org/wbos/default.aspx

[B16] Smith DH Gravelle H The practice of discounting in economic evaluations of healthcare interventions Int J Technol Assess Health Care 2001 17 236 243 10.1017/S0266462300105094 11446135

[B17] Graf von der Schulenburg JM Greiner W Jost F Klusen N Kubin M Leidl R Mittendorf T Rebscher H Schoeffski O Vauth C German recommendations on health economic evaluation: third and updated version of the Hanover Consensus Value Health 2008 11 539 544 10.1111/j.1524-4733.2007.00301.x 18194408

[B18] National Institute for Clinical Excellence (NICE) Guideline Development Methods: Information for National Collaborating Centres and Guideline Developers Book Guideline Development Methods: Information for National Collaborating Centres and Guideline Developers 2004 City: National Institute for Clinical Excellence (Editor ed.^eds.)

[B19] Dzator JA Hendrie D Burke V Gianguilio N Gillam HF Beilin LJ Houghton S A randomized trial of interactive group sessions achieved greater improvements in nutrition and physical activity at a tiny increase in cost J Clin Epidemiol 2004 57 610 619 10.1016/j.jclinepi.2003.10.009 15246129

[B20] Elley R Kerse N Arroll B Swinburn B Ashton T Robinson E Cost-effectiveness of physical activity counselling in general practice N Z Med J 2004 117 U1216 15608809

[B21] Proper KI de Bruyne MC Hildebrandt VH van der Beek AJ Meerding WJ van Mechelen W Costs, benefits and effectiveness of worksite physical activity counseling from the employer's perspective Scand J Work Environ Health 2004 30 36 46 15018027 10.5271/sjweh.763

[B22] Stevens W Hillsdon M Thorogood M McArdle D Cost-effectiveness of a primary care based physical activity intervention in 45-74 year old men and women: a randomised controlled trial Br J Sports Med 1998 32 236 241 10.1136/bjsm.32.3.236 9773174 PMC1756094

[B23] Munro JF Nicholl JP Brazier JE Davey R Cochrane T Cost effectiveness of a community based exercise programme in over 65 year olds: cluster randomised trial J Epidemiol Community Health 2004 58 1004 1010 10.1136/jech.2003.014225 15547060 PMC1732655

[B24] Finkelstein EA Troped PJ Will JC Palombo R Cost-effectiveness of a cardiovascular disease risk reduction program aimed at financially vulnerable women: the Massachusetts WISEWOMAN project J Womens Health Gend Based Med 2002 11 519 526 10.1089/152460902760277877 12243129

[B25] Robertson MC Devlin N Scuffham P Gardner MM Buchner DM Campbell AJ Economic evaluation of a community based exercise programme to prevent falls J Epidemiol Community Health 2001 55 600 606 10.1136/jech.55.8.600 11449021 PMC1731948

[B26] Robertson MC Gardner MM Devlin N McGee R Campbell AJ Effectiveness and economic evaluation of a nurse delivered home exercise programme to prevent falls. 2: Controlled trial in multiple centres BMJ 2001 322 701 704 10.1136/bmj.322.7288.701 11264207 PMC30095

[B27] Robertson MC Devlin N Gardner MM Campbell AJ Effectiveness and economic evaluation of a nurse delivered home exercise programme to prevent falls. 1: Randomised controlled trial BMJ 2001 322 697 701 10.1136/bmj.322.7288.697 11264206 PMC30094

[B28] Ackermann RT Williams B Nguyen HQ Berke EM Maciejewski ML LoGerfo JP Healthcare cost differences with participation in a community-based group physical activity benefit for medicare managed care health plan members J Am Geriatr Soc 2008 56 1459 1465 10.1111/j.1532-5415.2008.01804.x 18637982 PMC3036984

[B29] The Writing Group for Activity Counselling Trail Research Group Effects of physical activity counselling in primary care: the Activity Counselling Trial: a randomized controlled trial JAMA 2001 286 677 687 10.1001/jama.286.6.677 11495617

[B30] Shephard RJ Corey P Renzland P Cox M The impact of changes in fitness and lifestyle upon health care utilization Can J Public Health 1983 74 51 54 6406030

[B31] Shephard RJ Corey P Renzland P Cox M The influence of an employee fitness and lifestyle modification program upon medical care costs Can J Public Health 1982 73 259 263 6814742

[B32] Baun WB Bernacki EJ Tsai SP A preliminary investigation: effect of a corporate fitness program on absenteeism and health care cost J Occup Med 1986 28 18 22 10.1097/00043764-198601000-00007 3081697

[B33] Ackermann RT Cheadle A Sandhu N Madsen L Wagner EH LoGerfo JP Community exercise program use and changes in healthcare costs for older adults Am J Prev Med 2003 25 232 237 10.1016/S0749-3797(03)00196-X 14507530

[B34] Chen IJ Chou CL Yu S Cheng SP Health services utilization and cost utility analysis of a walking program for residential community elderly Nurs Econ 2008 26 263 269 18777976

[B35] Shephard RJ Long term impact of a fitness programme--the Canada Life Study Ann Acad Med Singapore 1992 21 63 68 1590660

[B36] Sevick MA Dunn AL Morrow MS Marcus BH Chen GJ Blair SN Cost-effectiveness of lifestyle and structured exercise interventions in sedentary adults: results of project ACTIVE Am J Prev Med 2000 19 1 8 10865157 10.1016/s0749-3797(00)00154-9

[B37] Brouwer W Rutten F Koopmanschap M Drummond MF, McGuire A Costing in economic evaluations Economic evaluations in Health Care - Merging theory with practice 2001 Oxford, New York Oxford University Press

[B38] Krauth C Hessel F Hansmeier T Wasem J Seitz R Schweikert B [Empirical standard costs for health economic evaluation in Germany - a proposal by the working group methods in health economic evaluation] Gesundheitswesen 2005 67 736 746 10.1055/s-2005-858698 16235143

[B39] Gold MR Russell LB Siegel JE Cost-effectiveness in health and medicine 1996 1 New York: Oxford University Press

[B40] Haycox A A methodology for estimating the costs and benefits of health promotion Health Prom Int 1994 9 5 11 10.1093/heapro/9.1.5

[B41] Drummond MF Sculpher M Torrance GW O'Brien BJ Stoddart GL Methods for Economic Evaluation of Health Care Programmes 2005 3 Oxford, New York Oxford University Press

[B42] Welte R Feenstra T Jager H Leidl R A decision chart for assessing and improving the transferability of economic evaluation results between countries PharmacoEcon 2004 22 857 876 10.2165/00019053-200422130-00004 15329031

[B43] The Writing Group for Activity Counselling Trail Research Group Effects of physical activity counseling in primary care: the Activity Counseling Trial: a randomized controlled trial The Journal of the American Medical Association 2001 286 677 687 10.1001/jama.286.6.677 11495617

